# Emotional Intelligence and Transformational Leadership: Meta-Analysis and Explanatory Model of Female Leadership Advantage

**DOI:** 10.3390/jintelligence10040104

**Published:** 2022-11-14

**Authors:** Ning Hsu, Daniel A. Newman, Katie L. Badura

**Affiliations:** 1 Department of Psychology, Virginia Polytechnic Institute and State University, Blacksburg, VA 24060, USA; 2 Department of Psychology, University of Illinois at Urbana-Champaign, Champaign, IL 61820, USA; 3 Scheller College of Business, Georgia Institute of Technology, Atlanta, GA 30308, USA

**Keywords:** emotional intelligence, female leadership advantage, transformational leadership, gender, agency, communion, meta-analysis

## Abstract

Emotional intelligence is a second-stratum factor of general intelligence (MacCann et al. 2014) that: (a) has been popularly touted as an essential individual difference for effective leadership (Goleman 1998), but also (b) exhibits large gender group differences favoring women (Joseph and Newman 2010). Combining these insights, we propose that emotional intelligence is a key mechanism in the so-called female leadership advantage (Eagly and Carli 2003—which emphasizes the finding that women are rated slightly higher in transformational leadership compared to men). The current study seeks to explain this gender leadership gap by specifying three personality-based theoretical mechanisms that enhance transformational leadership: (a) emotional intelligence (favoring women), (b) communion (stereotypical femininity; favoring women; Hsu et al. 2021), as well as an offsetting effect of (c) agency (stereotypical masculinity; favoring men). Meta-analytic data (including original meta-analyses among the leader’s ability-based emotional intelligence, transformational leadership, communion, and agency) are used to test our theorized model. Results confirm the full mediation model of female leadership advantage. Because the three unique mechanisms operate in different directions, their individual indirect effects are notable, but their cumulative indirect effect is small and near-zero. In conclusion, we emphasize incorporating emotional intelligence with other personality-based explanations of gender effects in leadership perceptions.

## 1. Introduction

The current work investigates the so-called *female leadership advantage*, with the goal of offering a theoretical model to explain the personality and individual difference origins of gendered leadership advantages. In particular, the model we propose here differs from previous work on personality-based models of gender gaps in leadership (Badura et al. 2018), because we emphasize the incorporation of one underappreciated explanatory mechanism—ability-based emotional intelligence (Mayer and Salovey 1997). Ability-based emotional intelligence is a second-stratum factor of human cognitive ability, reflecting intelligence in the emotion domain (MacCann et al. 2014). In short, we specify and test a model asserting that women are perceived as more transformational leaders in part because they exhibit much stronger ability-based emotional intelligence than men, on average.

En route to demonstrating how ability-based emotional intelligence can play a unique role in the female leadership advantage, we will: (a) summarize the nuanced evidence for the female leadership advantage, with an emphasis on transformational leadership, and (b) propose and test an integrated personality-based model of the female leadership advantage, specifying the relative roles of three attributes (emotional intelligence, communion, and agency) in explaining the gender gap in transformational leadership. In order to test the model, we provide updated meta-analyses of the relationships between ability-based emotional intelligence, transformational leadership, communion, and agency.

### 1.1. Female Leadership Advantage

When thinking of great leaders throughout history, many people might think of Mahatma Gandhi, Abraham Lincoln, Nelson Mandela, and Winston Churchill, to name but a few. One thing these leaders have in common is that they all happen to be of one gender: male. Indeed, the historical prototype or stereotype of a great (or good) leader has typically been male and masculine (Koenig et al. 2011). Such masculine leader stereotypes could also create a disadvantage for women seeking or performing in leadership roles, such that it is more difficult for women to become or be perceived as a good leader, due to the mismatch or incongruity between the role expectations for women and the role expectations for leaders (Eagly and Karau 2002). Indeed, various discrimination-based theories of gender gaps in leadership attainment and leader evaluations have received a great deal of attention (i.e., Think Manager—Think Male: Schein 1973; Role Congruity Theory: Eagly and Karau 2002; Lack of Fit Theory: Heilman 2001), as these theories are typically used to explain the underrepresentation or undervaluing of women in leadership roles (i.e., female leadership disadvantage).

However, the contention that women might, in fact, have a leadership *advantage* in contemporary society has also been popularized in recent years. One version of the female leadership advantage perspective (cf. Eagly and Carli 2003; Vecchio 2002; Yukl 2002) proposes that women have an advantage in leadership because women have more skills and experiences in interpersonal relationships, inclusive decision making, caregiving, and power-sharing (Grant 1988; also see Lipman-Blumen 1983; Vecchio 2002). When authors (e.g., Eagly and Carli 2003; Eagly 2007) refer to the female leadership advantage, they often cite meta-analytic empirical evidence of various kinds. This evidence is briefly reviewed below.

#### 1.1.1. Gender Differences in Leadership Style

Eagly and Johnson (1990) meta-analyzed the gender differences in various leadership styles, including interpersonal style, task style, interpersonal-task style continuum, and democratic-autocratic style continuum. Task style refers to a leadership style with an orientation to task accomplishment, such as coordinating activities to complete assigned tasks; whereas interpersonal style refers to a leadership style with a socioemotional orientation, focusing on the maintenance of interpersonal relationships as well as the morale and welfare of the group members (Bales 1950). Task style and interpersonal style were most famously developed in the Ohio State studies of leader behavior, where these two factors are, respectively, known as *initiating structure* and *consideration* (e.g., Halpin 1957; Halpin and Winer 1957; Hemphill and Coons 1957; Stogdill 1963). Eagly and Johnson (1990) found that on average, women exhibited very slightly higher interpersonal style than men did (*d* = .04, CI [.01, .07], *k* = 136), while women exhibited an identical level of task style as men did (*d* = .00, CI [−.03, .03], *k* = 139). Relatedly, when meta-analyzing the set of primary studies that measured interpersonal style and task style on a single interpersonal-task style continuum, men and women scored similarly (*d* = −.03, CI [−.10, .03], *k* = 31). The largest gender effect found in Eagly and Johnson’s (1990) meta-analysis demonstrated that women exhibited a more democratic style of leadership (on the democratic-autocratic style continuum) than men on average (*d* = .22, CI [.15, .29], *k* = 23; Eagly and Johnson 1990). In general, however, the gender gaps in leadership styles found in Eagly and Johnson’s meta-analysis were almost always near-zero.

The construct validity of Eagly and Johnson’s (1990) meta-analysis has also raised concerns (Vecchio 2002). First, the gender effects were averages across a wide range of measures, including the Leader Behavior Description Questionnaire (LBDQ; Stogdill 1963; Stogdill et al. 1962), Leadership Effectiveness and Adaptability Description (LEAD; Hersey and Blanchard 1977, 1982), Leadership Opinion Questionnaire (Fleishman 1953, 1957, 1960), Organizational Climate Description Questionnaire (Halpin 1966), and so on; with these instruments all designed to assess different constructs. Eagly and Johnson’s (1990) meta-analysis also included measures such as the Least Preferred Co-Worker scale (Fiedler 1967) that have not shown adequate convergent validity with more rigorously validated and established measures of leadership style (e.g., LBDQ; Stogdill 1963; Stogdill et al. 1962). Second, this meta-analysis was largely based on leaders’ self-ratings (197 out of the total 370 primary studies included in the meta-analysis used leaders’ self-ratings of leadership), and Eagly and Johnson (1990) did not report the gender effects separately by rating source (e.g., reporting gender effects for self-rating vs. other-rating separately). Therefore, it is possible the results from Eagly and Johnson (1990) could have been largely driven by leaders’ self-stereotype or social desirability, instead of observed leadership behaviors (as observed by others).

Along the same lines, a more recent meta-analysis on paternalistic leadership style (Hiller et al. 2019) shows that the gender gaps in authoritarianism (*r* = −.01, *ρ* = −.01, CI [−.06, .04], *k* = 11, *N* = 4385), benevolence (*r* = −.01, *ρ* = −.01, CI [−.04, .01], *k* = 11, *N* = 5236), and morality (*r* = .03, *ρ* = .03, CI [.00, .07], *k* = 7, *N* = 2754) were also close to zero. Altogether, this evidence supports the contention that the gender differences in leadership styles may be negligible and near-zero.

#### 1.1.2. Gender Differences in Leadership Effectiveness

Besides the gender differences in leadership styles, research has also looked into whether there is a gender difference in leadership effectiveness (e.g., Paustian-Underdahl et al. 2014). Measures of leadership effectiveness included in Paustian-Underdahl et al.’s (2014) meta-analysis consist of: (a) leader performance, (b) leadership ability, (c) measures of satisfaction with leaders, (d) measures of satisfaction with leaders’ performance, (e) coding/counting of effective leadership behaviors, and (f) evaluations of organizational productivity/team performance. Paustian-Underdahl et al.’s (2014) meta-analysis found that women are perceived to be slightly, but non-significantly, more effective leaders than men (overall *d* = −.05, *k* = 99, *N* = 101,676). Importantly, this gender difference is larger and statistically significant when looking at only other-ratings of leader effectiveness (other-rating *d* = −.12, *k* = 78, *N* = 96,893). Further, when looking at only self-ratings of leader effectiveness, male leaders exhibited a higher mean than female leaders (self-rating *d* = .21, *k* = 19, *N* = 4711). In other words, the direction of the gender gap in leader effectiveness ratings depended entirely on whether the leader effectiveness was self-reported. However, the construct validity of Paustian-Underdahl et al.’s (2014) meta-analysis is a potential source of ambiguity (the same as in all past meta-analyses of leadership effectiveness), because there is no standardized measurement of leadership effectiveness typically used in the literature.

#### 1.1.3. Gender Differences in Transformational Leadership

Perhaps the best evidence for an actual female leadership advantage comes from the domain of transformational leadership (Eagly et al. 2003). Transformational leaders are defined as leaders who give vision and sense of mission, earn respect and trust (charisma), convey high expectations and communicate purposes effectively (inspiration), encourage attentive thinking and problem solving (intellectual stimulation), and offer individual attention and mentor followers individually (individualized consideration; Bass 1990). There are several potential reasons why transformational leadership style would be enacted or perceived to be enacted especially well by women (cf. Eagly and Carli 2003; Yoder 2001). First, transformational leadership behaviors, unlike more traditionally emphasized leadership behaviors, have sometimes been thought of as more stereotypically androgynous and/or feminine (not exclusively masculine; Eagly and Carli 2003). This characteristic of transformational leadership allows women to be more freed from the double-bind created by the role incongruity between their stereotypical gender roles and their leader roles. Second, women might be especially adept at executing the more stereotypically feminine facets of transformational leadership (e.g., individualized consideration) because of a spillover effect from their gender roles to their leader roles. Third, women who survived the double standard and glass ceiling in leader selection in organizations might possess more leadership skills than their male counterparts (who did not face the same hurdles in order to be in a leadership position; Eagly et al. 2003; see also Eagly and Carli 2003).

In support of this view, Eagly et al.’s (2003) meta-analysis of gender and transformational leadership found that, on average, women were rated higher on transformational leadership than men were (*d* = −.10, *k* = 44). This finding of a female leadership advantage was supported across different measures of transformational leadership: MLQ norming study (*d* = −.11); other MLQ studies (*d* = −.11, *k* = 26); and studies using other measures (*d* = −.09, *k* = 17). Importantly, these gender effects are larger when only looking at subordinate ratings (*d* = −.15, *k* = 26) compared to leader’s self-ratings of their own transformational leadership (*d* = −.06, *k* = 26). Further, some evidence supports measurement equivalence between men and women for transformational leadership ratings (Walumbwa et al. 2005), similar to various other types of leadership (e.g., assessment center leadership ratings, Anderson et al. 2006; authentic leadership, Caza et al. 2010). Such evidence suggests the mean difference between men and women in transformational leadership should largely reflect a true difference in the latent construct of transformational leadership, rather than a measurement artifact.

Due to the pattern of empirical findings just reviewed, in the current study we will focus on the gender gap in transformational leadership, as that is the domain where the female leadership advantage has been most clearly demonstrated (Eagly and Carli 2003). Specifically, we will begin by focusing on transformational leadership measures that are not self-reported (i.e., separating self-rated transformational leadership from non-self-rated transformational leadership). Our initial focus on other-rated leadership is consistent with common definitions of leadership that emphasize how leaders influence their followers and others (e.g., Winston and Patterson 2006; Silva 2016).

### 1.2. Personality-Based Leadership Theories

The theory of leadership categorization (Lord et al. 1984) proposes that people have *implicit theories of leadership*, such that they possess certain ideas of what most leaders are like (prototypes). For example, people find specific traits (e.g., charisma, masculinity, etc.) to characterize their cognitive schema for leaders (Offermann et al. 1994). This conceptualization of personality and leadership (Lord et al. 1986) is rooted in a social-cognitive perspective (e.g., Mischel 1973), which suggests that personality traits should influence social perceptions because observers tend to use others’ personality traits to help organize their perceptions of others (Winter and Uleman 1984). According to this view, the personality traits of leaders should affect how they are perceived as a leader. Zaccaro (2007) further proposed an integrated model where attributes (e.g., personality and cognitive abilities, social appraisal and problem-solving skills) operate through leader processes to predict leadership outcomes (i.e., leader emergence, leader effectiveness, and leader advancement and promotion). Based on the theory of leadership categorization (Lord et al. 1984) and a model of leader attributes and leader performance (Zaccaro 2007), personality traits could be one potential reason that helps explain the *female leadership advantage*—i.e., the observed gender gap in transformational leadership. In our attempt to explain the female leadership advantage, we focus on three traits/individual differences in particular: emotional intelligence, communion, and agency.

#### 1.2.1. Emotional Intelligence and Leadership

Goleman’s (1995, 1996, 1998; Goleman et al. 2001) best-selling books and most-downloaded articles have fueled the popularization of emotional intelligence. One reason for the popularity of this work is Goleman’s (1998) expansive claim that “IQ and technical skills are important, but emotional intelligence is the sine qua non of leadership” (p. 82)—i.e., the claim that emotional intelligence is an essential individual attribute for being a great leader. To elaborate, Goleman (1998) advanced several components of emotional intelligence at work—including self-regulation, empathy, and social skill—to advance his conceptual (and non-empirical) explanation for his claim that emotional intelligence matters more than general cognitive ability/intelligence. To begin, cognitive function is worse when people are emotionally upset or disregulated. Further, emotional intelligence helps people to be more perceptive and attentive to the emotions of others, to be able to manage conflict, to be able to communicate in a more constructive and helpful way, to be able to help build a climate where diversity is valued instead of a source of conflict, and to be able to collaborate with others more effectively (Goleman 1996).

Empirically, emotional intelligence (EI) has been defined and measured in two ways (Mayer et al. 2000): (a) ability EI, and (b) mixed EI. Ability EI was defined as the ability to understand emotions and apply emotions and emotional knowledge to improve the thinking process (Mayer et al. 2008), whereas mixed EI was defined (more broadly) as noncognitive ability or skill (Baron 1997), socially or emotionally intelligent behaviors (Bar-On 2004), and emotion-related personality traits (Petrides and Furnham 2003). Much of the criticism of EI has focused on mixed EI because of its broad definition (EI as “a grabbag of everything that is not cognitive ability”; Joseph and Newman 2010, p. 72; Murphy 2006) and its lack of discriminant validity from personality traits (de Raad 2005). Indeed, meta-analysis shows that mixed EI is largely redundant with a hodgepodge of other well-known traits including: (a) ability EI, (b) self-efficacy, (c) self-rated performance, (d) conscientiousness, (e) emotional stability, (f) extraversion, and (g) general mental ability (Joseph et al. 2015).

In contrast, ability EI has been argued to have a clear theoretical definition and to therefore be the only conceptualization of EI worthy of future study (Daus and Ashkanasy 2005). In support of ability EI, MacCann et al. (2014) demonstrated empirically that ability EI fits well within the general hierarchical structure of human cognitive abilities as a second-stratum factor that marks “the expression of intelligence in the emotion domain” (p. 358). Ability EI is commonly understood using the Four-Branch Model of EI (Mayer and Salovey 1997; Salovey and Mayer 1990), where ability EI consists of (a) perceiving emotion accurately, (b) utilizing emotions to improve thinking processes, (c) making sense of emotions, and (d) regulating emotions. Given its superior conceptualization as a facet of actual intelligence, the current study will focus exclusively on the ability EI definition (i.e., EI measured using performance-based measures—measures that exclusively employ right-wrong and/or multiple-choice formats, with these tests of ability EI administered to the leaders themselves).

George (2000) further proposed that the four components of ability EI contribute to effective leadership. She defined effective leadership using many of the features of transformational leadership, including (a) providing vision, (b) stimulating intelligence, (c) motivating confidence, (d) encouraging flexibility, and (e) giving meaning; and she argued that leaders high in EI would be better equipped with the socioemotional skills to enact these effective leadership behaviors, because these behaviors are emotion-driven. Echoing this logic, we propose that ability EI will positively predict transformational leadership.

**Hypothesis** **1** **(H1).** *Emotional intelligence has a positive relationship with transformational leadership (i.e., this hypothesis involves ability-based EI).*

#### 1.2.2. Emotional Intelligence and Gender

Given that a major objective of the current study is to explain the mechanisms underlying the female leadership advantage, we note that ability-based EI—which we hypothesized to predict transformational leadership—is also known to exhibit a large gender gap favoring women (Joseph and Newman 2010). When explaining gender gaps in personality and individual differences (like the observed gap in ability EI), a useful theoretical framework is social role theory (Eagly 1987; Wood and Eagly 2012). According to social role theory, gender differences in individual attributes are rooted in the gendered division of labor. Specifically, biological specialization (related to men’s advantage in average upper body strength and women’s advantage in childcare/nursing) gives rise to gender segregation in jobs/roles, and this gendered division of labor forms a basis for dispositional inferences about gender group differences in traits. Social role expectations and gender norms are then reinforced via the socialization of internal gender identities (Heilman 1983; Eagly and Wood 2009), as well as social regulation, external pressure or backlash against exhibiting gender-non-normative attributes (Rudman et al. 2012; Williams and Tiedens 2016; see review by Hsu et al. 2021).

What are the implications of social role theory for the gender gap in emotional intelligence? To recap, social role theory holds that gendered socialization pressures tend to correspond to the gendered division of labor in a given society (Wood and Eagly 2002). As a result of the general tendency toward occupational gender segregation (concentration of women in “domestic work and communally demanding employment”—e.g., nursing, teaching, childcare, and other people-oriented occupations; (Su et al. 2009), observers often “infer that [women] are warm, caring, and socially skilled” (Wood and Eagly 2012, p. 71). However, we contend that these gendered dispositional inferences about women can be further parsed into two major individual difference components: (a) motivational/discretionary (“will do”) attributes, and (b) skill- and ability-based (“can do”) attributes. The prior category (i.e., gendered “will do” attributes) coalesces around the broad personality trait of communion (see Bem 1974; described in more detail below); while in the latter category (i.e., gendered “can do” attributes) coalesces around the set of *knowledge*, *skills*, and *abilities* that facilitate the achievement of communal goals (e.g., nurturance, warmth, empathy). Importantly, we contend that this set of gender-normed individual differences in knowledge, skill, and ability that facilitate communal goals is well-encapsulated by the concept of ability-based emotional intelligence. In support of the view that women are socialized into higher mean levels of emotional intelligence (e.g., emotion perception, emotion understanding, and emotion regulation) in order to accommodate gendered social roles that are often prescribed by the division of labor, Joseph and Newman (2010) found that women scored substantially higher on tests of ability-based emotional intelligence than did men (ability-based EI *d*_corrected_ = .52, *k* = 14, *N* = 2216).

As mentioned earlier, meta-analysis has estimated that women exhibit slightly more transformational leadership than men, on average (*d* = −.10, *k* = 44; Eagly et al. 2003). Eagly et al. (2003) contend that one reason why women are more transformational leaders is because women are more communal. However, they did not look at socio-emotional skills. As such, we offer a novel and complementary potential explanation for the female transformational leadership advantage—emotional intelligence. Transformational leadership involves more socio-emotional aspects of leadership; thus, women’s higher emotional intelligence is likely one reason why there is a slight female advantage in transformational leadership.

**Hypothesis** **2** **(H2).** *Emotional intelligence mediates the effect of gender (favoring women) on transformational leadership.*

#### 1.2.3. Agency and Communion

In discussions of gender and personality, much of the theoretical and research attention has involved the two concepts of communion and agency (Bakan 1966; Wiggins 1991). Communion is a female-associated trait (also called femininity), while agency is a male-associated trait (also called masculinity), although each of the two traits can be exhibited by both men and women alike (Bem 1974; Spence and Helmreich 1978; also see meta-analytic review by Hsu et al. 2021). The tradition of studying gender identity via gender-stereotypical personality traits became the basis for numerous research studies, in which communion was characterized using terms such as warmth, affection, and sympathy; while agency was characterized using terms such as assertiveness, forcefulness, and self-reliance (Wood and Eagly 2015; cf. Ma et al. 2022).

Traditionally, agency has been an advantageous trait for leaders to possess. For example, agency (or masculinity) has been found empirically to be one of the strongest predictors of both leadership emergence and leadership effectiveness (Lord et al. 1986; Mann 1959), and this finding has been echoed in research on the related trait of dominance (Hogan 1978; Judge et al. 2002; Smith and Foti 1998; Stogdill 1948). Indeed, one classic study (Megargee 1969) showed in a lab experiment that the high-dominance individual in a mixed-dominance dyad tended to emerge as the leader, unless the high-dominance individual was a woman paired with a low-dominance man, in which case the man was chosen as leader. A more recent meta-analysis (Badura et al. 2018) has shown that: (a) women are less likely to be chosen as a leader, and (b) this gender gap in leader emergence can be largely explained by agency. In other words, agentic personality can serve as a mediator for gender effects on leadership.

Wiggins (1991) argues that both agentic (i.e., getting ahead) and communal (i.e., getting along) traits are essential for individuals to be able to function well in human society. An individual needs to be agentic enough in order to be in charge and be able to protect their own resources for survival, while they need to be communal enough in order to maintain relationships, be popular and be able to avoid sabotage from other people. As such, being agentic and being communal could both be functional. Similarly, we contend that both traits can be functional in being perceived as an effective leader. However, agency and communion are gendered traits that have consistently shown mean gender differences across time. A recent meta-analysis has shown that, on average, men are more agentic than women (*g* = .40, *k* = 928, *N* = 254,731) and women are more communal than men (*g* = −.56, *k* = 937, *N* = 254,465; Hsu et al. 2021). Therefore, gendered traits such as agency and communion could underlie offsetting gender-leadership effects, which would help explain the slight gender gap in transformational leadership favoring women. That is, the agentic leadership advantage for men and the communal leadership advantage for women could largely cancel each other out, resulting in a very slight female leadership advantage (Eagly et al. 2003). Altogether, based on the circumplex of interpersonal variables (Wiggins 1991), we propose that both agency and communion would have a positive relationship with transformational leadership, and that both agency and communion would mediate the effect of gender on transformational leadership, albeit in opposite directions.

**Hypothesis** **3** **(H3).** *Communal traits have a positive relationship with transformational leadership.*

**Hypothesis** **4** **(H4).** *Communal traits mediate an effect of gender (favoring women) on transformational leadership.*

**Hypothesis** **5** **(H5).** *Agentic traits have a positive relationship with transformational leadership.*

**Hypothesis** **6** **(H6).** *Agentic traits mediate an effect of gender (favoring men) on transformational leadership.*

### 1.3. Integrated Model of Female Leadership Advantage

**Figure 1:**
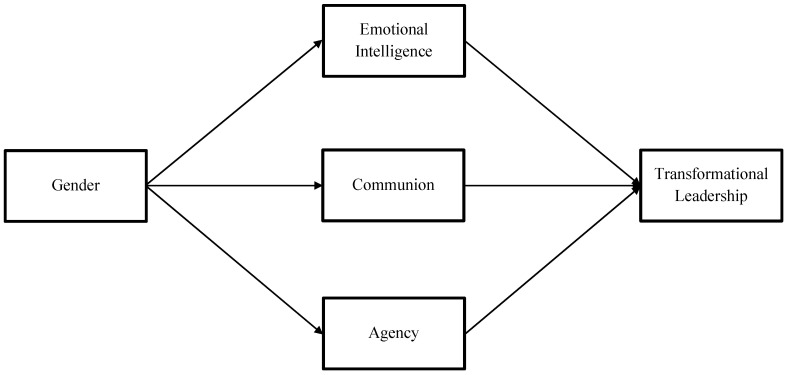
A Personality-Based Model of Female Leadership Advantage.

Finally, by combining the predictions specified in Hypotheses 1 through 6, we propose an integrated personality-based model of female leadership advantage (see Figure 1). In this model, we highlight the role of ability-based emotional intelligence as a mechanism for the female leadership advantage. We also emphasize that the model specifies three distinct mechanisms of gendered leadership advantage, which operate in differing directions: (a) a female leadership advantage via emotional intelligence, (b) a female leadership advantage via communion, and (c) a male leadership advantage via agency. It is the fact that these effects operate in opposite directions that produces a combined cumulative female leadership advantage that is small in magnitude.

## 2. Materials and Methods

In order to test the integrated theoretical model of the gender gap in transformational leadership (Figure 1), we combined meta-analytic correlations into an overall correlation matrix, which then served as the basis for path analysis (i.e., meta-analytic SEM; Viswesvaran and Ones 1995). In order to compile the overall meta-analytic correlation matrix, we used a combination of published meta-analyses and original meta-analyses.

### 2.1. Original Meta-Analyses

For relevant bivariate relationships that have already been meta-analyzed, we extracted effect sizes directly from previous meta-analyses (see Table 1). We also update the Harms and Credé (2010) meta-analysis when estimating the relationship between ability-based EI and transformational leadership measured from a different source (for which they reported *k* = 4 primary studies). We focus exclusively here on ability-based EI measured on the leader. To estimate this and other bivariate relationships that had not been previously meta-analyzed, we conducted eight original meta-analyses in the current study (using the Schmidt and Hunter 2015, approach). These relationships include (a, b) performance-based ability emotional intelligence with agency and communion, (c, d, e, and f) transformational leadership [both self-rated and non-self-rated] with agency and communion, and (g, h) transformational leadership [both self-rated and non-self-rated] with performance-based ability emotional intelligence.

#### 2.1.1. Literature Search

When conducting meta-analysis, there are two main approaches to addressing construct validity. One approach (the “kitchen sink” approach) is to include a set of primary studies that employ a wide range of both validated and unvalidated measures, as long as some claim can be generally made that each measure roughly assesses the target construct of interest (e.g., Barrick and Mount 1991). A second approach is to focus more exclusively on only primary studies that used validated measures of the construct of interest (e.g., Hurtz and Donovan 2000). In the current study, we chose to employ the latter approach. As such, to identify primary studies to include in our original meta-analyses, we searched within papers that cited the commonly used, validated measures of the constructs of interest. These primary studies were located using the Google Scholar ‘search within citing articles’ function.

For the relationships of emotional intelligence with agency and communion, we used the keywords *“MSCEIT” OR “MEIS” OR “STEU”* (i.e., the names of commonly used performance-based EI measures; to avoid the common term *STEM*, we only used *STEU* as one of our keywords), while searching within papers that cited commonly used and validated agency and communion measures (the Bem Sex Role Inventory—BSRI; Bem 1974; and personal attributes questionnaire—PAQ; Spence and Helmreich 1978; Spence et al. 1974, 1975), as done in previous meta-analyses (Hsu et al. 2021; Badura et al. 2018; Twenge 1997). For the relationships of transformational leadership with agency and communion, we used the keywords *“Multifactor Leadership Questionnaire” OR “Global Transformational Leadership” OR “Leadership Practices Inventory” OR “Transformational Leadership Inventory”* (i.e., the names of the most commonly used transformational leadership measures) to search within papers that cited commonly used, validated measures of agency and communion (BSRI; Bem 1974 and PAQ; Spence and Helmreich 1978; Spence et al. 1974, 1975). For the relationship between transformational leadership and performance-based emotional intelligence, we used the keywords *“MSCEIT” OR “MEIS” OR “STEU”* (i.e., the names of commonly used performance-based EI measures) to search within papers citing commonly used, validated transformational leadership measures, including the Multifactor Leadership Questionnaire (MLQ; Bass 1985), Global Transformational Leadership scale (GTL; Carless et al. 2000), Leadership Practices Inventory (LPI; Posner and Kouzes 1988), and Transformational Leadership Inventory (TLI; Podsakoff et al. 1990). Overall, 845 results were returned from the above keyword searches for papers that cited seminal works (i.e., Bem 1974; Spence and Helmreich 1978; Spence et al. 1974, 1975; Bass 1985; Carless et al. 2000; Posner and Kouzes 1988; Podsakoff et al. 1990).

#### 2.1.2. Inclusion Criteria

Studies were included in the meta-analysis according to the following rules. First, a primary study had to report sufficient statistics to calculate an effect size (e.g., correlation coefficient *r*) for one of the proposed relationships in the current study. Second, the samples in the primary studies needed to be adult samples (e.g., undergraduate samples and working adults). Third, only primary studies written in English were included in the current meta-analysis.

**Figure 2:**
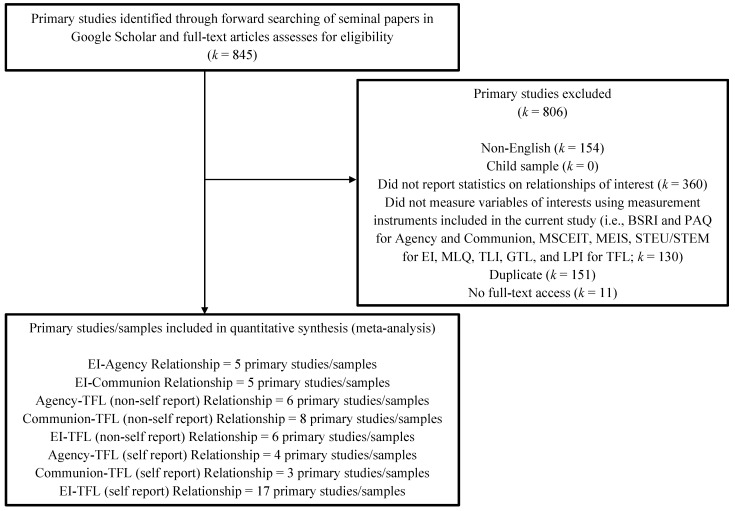
Flow Chart for Meta-Analytic Literature Search.

For the relationships involving emotional intelligence (i.e., the relationships of emotional intelligence with agency, communion, and transformational leadership), a primary study had to specifically measure the leaders’ EI using a performance-based ability EI measure (i.e., the MSCEIT; Mayer et al. 2003, MEIS; Mayer et al. 1999, and STEU/STEM; MacCann and Roberts 2008). That is, the leader needed to have taken a test of performance-based ability EI (i.e., a multiple-choice test that contains right and wrong response options). For the relationships involving transformational leadership (i.e., the relationships of transformational leadership with agency, communion, and emotional intelligence), a primary study had to measure transformational leadership using one of the validated transformational leadership measures, including the Multifactor Leadership Questionnaire (MLQ; Bass 1985), Global Transformational Leadership scale (GTL; Carless et al. 2000), Leadership Practices Inventory (LPI; Posner and Kouzes 1988), and Transformational Leadership Inventory (TLI; Podsakoff et al. 1990). Applying these criteria resulted in the inclusion of five effect sizes for the EI-Agency relationship (*N* = 1172), five effect sizes for the EI-Communion relationship (*N* = 1172), six effect sizes for the Agency-TFL (non-self report) relationship (*N* = 420), eight effect sizes for the Communion-TFL (non-self report) relationship (*N* = 779), six effect sizes for the EI-TFL (non-self report) relationship (*N* = 618), four effect sizes for the Agency-TFL (self report) relationship (*N* = 820), three effect sizes for the Communion-TFL (self report) relationship (*N* = 589), and 17 effect sizes for the EI-TFL (self report) relationship (*N* = 1923). A summary of the literature searches and exclusion/inclusion process (which adheres to PRISMA and APA guidelines) can be seen in Figure 2.

## 3. Results

### 3.1. Eight Original Meta-Analyses

Meta-analytic results for the EI-TFL relationship are reported in Table 2. Supporting Hypothesis 1, EI was positively related to TFL (non-self-reported): (*r* = .12, *ρ* = .13, *k* = 6, *N* = 618, 95% CI [.04, .20]). When looking at self-reported transformational leadership, EI was positively related to self-reported TFL (*r* = .25, *ρ* = .29, *k* = 17, *N* = 1923, 95% CI [.08, .50]). Meta-analytic results for the communion-TFL and agency-TFL relationships are reported in Table 3 and Table 4, respectively. Supporting Hypothesis 3, communion was positively related to TFL (non-self-reported): (*r* = .44, *ρ* = .50, *k* = 8, *N* = 779, 95% CI [.32, .57]). Agency had a positive, but not statistically significant relationship with TFL (non-self-reported): (*r* = .20, *ρ* = .27, *k* = 6, *N* = 420, 95% CI [−.06, .45]). When looking at self-reported transformational leadership, both communion (*r* = .30, *ρ* = .36, *k* = 3, *N* = 589, 95% CI [.11, .50]) and agency (*r* = .25, *ρ* = .31, *k* = 4, *N* = 820, 95% CI [.11, .39]) were positively related to self-reported TFL. Meta-analytic results for the communion-EI and agency-EI relationships are reported in Table 5. Both communion (*r* = .05, *ρ* = .07, *k* = 5, *N* = 1172, 95% CI [−.06, .16]) and agency (r = −.0009, *ρ* = −.0005, k = 5, N = 1172, 95% CI [−.05, .05]) did not have statistically significant relationships with ability-based EI.

### 3.2. Moderator Analyses

When testing continuous moderators (i.e., date of study and age of sample), we found no statistically significant results (all *p* > .05; *n.s.*). Further, when testing categorical moderators, we only interpreted moderators when at least 3 primary studies were available at each level of the moderator (see Table 2, Table 3 and Table 4). Only 4 moderator tests were statistically significant. We found that the EI-TFL relationship was weaker when TFL was rated by others (*r* = .12, *ρ* = .13), compared to when TFL was rated by the leaders themselves (*r* = .25, *ρ* = .29; Δ*_ρ_* = .16; 95% CI [.02, .26], *p* < .05; see Table 2). The communion-TFL relationship was weaker in US samples (*r* = .27, *ρ* = .31), compared to non-US samples (*r* = .47, *ρ* = .53; Δ*_ρ_* = −.22, 95% CI [−.47, −.01], *p* < .05; see Table 3), while the agency-TFL relationship was stronger in US samples (*r* = .36, *ρ* = .43), compared to non-US samples (*r* = .10, *ρ* = .13; Δ*_ρ_* = .30, 95% CI [.10, .54], *p* < .05; see Table 4). Finally, the agency-TFL relationship was weaker in published papers (*r* = .15, *ρ* = .21), versus unpublished papers (*r* = .36, *ρ* = .43; Δ*_ρ_* = .22, 95% CI [.06, .47], *p* < .05; see Table 4).

### 3.3. Mediator Analyses

To test the hypothesized mediating relationships, we conducted path analyses in LISREL 10.1 using the meta-analytic correlation matrix shown in Table 1, with the minimum sample size *N*. We chose to use the minimum sample size when running the path analyses in order to be conservative (Badura et al. 2020), because of the considerable difference in the sample sizes among the meta-analytic correlations, and the fact that several of the proposed mediator bivariate relationships had modest sample sizes (ranging from 420 to 1923; see Table 1). As a supplemental analysis, all path analyses and mediation tests were also run using the harmonic mean *N*, and the direction and statistical significance of the results were identical to the results reported here using the minimum *N*. Six theoretical models were run and compared (Model 1a compared with 1b, and 2a compared with 2b, 2c, and 2d; see Table 6). Model comparisons were made on the basis of change in CFI, with a model difference being interpreted as meaningful when ΔCFI > .01 (Cheung and Rensvold 2002).

**Figure 3:**
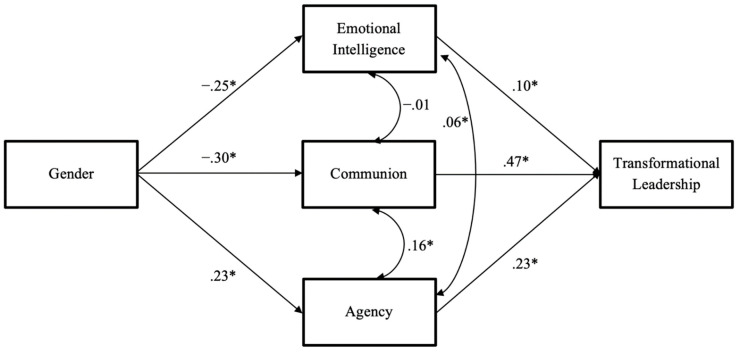
Path Model Results for the Personality-Based Model of Female Leadership Advantage (Model 1a). Note. Standardized path coefficients (*β*’s) are presented. *N* = 420; χ^2^ (1) = .434; RMSEA = .000, CFI = 1.000; NNFI = 1.023; SRMR = .006; * *p* < .05. Transformational leadership non-self-reported. Female = 1, Male = 2.

The hypothesized full mediation model (Model 1a: shown in Figure 1) displayed good overall fit (*χ*^2^_(df=1)_ = 1.327, RMSEA = .016, CFI = 1.00, NNFI = .996, SRMR = .006). Model 1b (partial mediation, including the direct effect from gender to TFL) was a saturated model with zero degrees of freedom, and therefore displayed perfect fit by design. We compared Model 1a against Model 1b using change in CFI (Cheung and Rensvold 2002) and retained Model 1a as the better model. Path coefficients for Model 1a are shown in Figure 3. All hypothesized paths were statistically significant in the expected directions.

**Figure 4:**
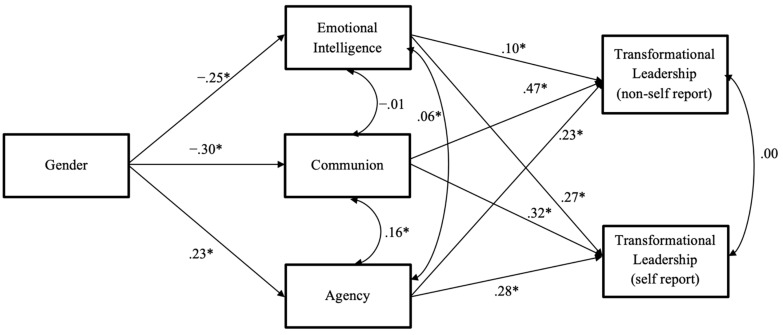
Path Model Results for the Personality-Based Model of Female Leadership Advantage (Model 2a). Note. Standardized path coefficients (*β*’s) are presented. *N* = 420; χ^2^ (2) = 2.513; RMSEA = .025, CFI = .999; NNFI = .990; SRMR = .013; * *p* < .05. Female = 1, Male = 2.

For the model comparisons among Models 2a, 2b, 2c, and 2d (models including both transformational leadership (non-self report) and transformational leadership (self report) as 2 DVs), Model 2a (hypothesized full mediation model: shown in Figure 4) displayed adequate overall fit (*χ*^2^_(df=2)_ = 11.517, RMSEA = .0384, CFI = .995, NNFI = .961, SRMR = .013). We next specified a series of partial mediation models, for the sake of model comparison. Model 2b (adding a direct path from gender to non-self-reported TFL) displayed adequate overall fit (*χ*^2^_(df=1)_ = 9.503, RMSEA = .0665, CFI = .995, NNFI = .930, SRMR = .012), as did Model 2c (adding a direct path from gender to self-reported TFL; *χ*^2^_(df=1)_ = 1.990, RMSEA = .0227, CFI = .999, NNFI = .992, SRMR = .0052). Model 2d (saturated model, with both direct paths—from gender to self- and non-self-reported TFL) displayed perfect fit by design (zero degrees of freedom). When comparing Models 2a, 2b, 2c, and 2d using change in CFI, we retained Model 2a as the best fitting model. Path coefficients for Model 2a are shown in Figure 4. All paths were statistically significant in the expected directions.

To test the indirect effects specified in the mediation hypotheses (H2, H4, and H6), we next used Monte Carlo confidence intervals (Preacher and Selig 2012; see Table 7). In particular, we tested whether the product of the path coefficients of X → M (e.g., Gender → EI) and M → Y (e.g., EI → TFL) was statistically significant. As shown in Table 7, three indirect effects: (a) Gender → EI → TFL, (b) Gender → Communion → TFL, and (c) Gender → Agency → TFL were tested, and all three were statistically significant—suggesting that EI, communion, and agency jointly mediate the relationship between gender and transformational leadership (supporting H2, H4, and H6, as well as the overall hypothesized model shown in Figure 1).

## 4. Discussion

There has been growing academic discussion of the *female leadership advantage* over the past decades (Eagly and Carli 2003; Paustian-Underdahl et al. 2014; Vecchio 2002; Yukl 2002), largely motivated by the finding of a small advantage for women in non-self-reported measures of transformational leadership (Eagly et al. 2003). However, it is unclear why women might be better transformational leaders compared to men. Therefore, one main goal of the current study was to identify and test potential personality-based individual differences that might give women an advantage in leadership.

Results confirm two, offsetting sets of phenomena. First, emotional intelligence and communal personality (i.e., stereotypical femininity, marked by warmth and compassion) both help women to be perceived as better leaders than men. Second, agentic personality (i.e., stereotypical masculinity, marked by assertiveness and dominance) helps men to be perceived as better leaders than women. These three mechanisms (emotional intelligence, communion, and agency) operate simultaneously and result in a near-zero cumulative gender effect on transformational leadership.

### 4.1. Theoretical Contributions

Although the idea of female leadership advantage (Paustian-Underdahl et al. 2014; cf. Eagly and Carli 2003; Vecchio 2002; Yukl 2002; Grant 1988; also see Lipman-Blumen 1983; Vecchio 2002) has been increasingly noted in recent decades, many of the classic and better-known theories of gender gaps in leadership attainment and leader evaluations—those that favor men—have continued to receive a great deal of attention (i.e., Think Manager—Think Male: Schein 1973; Role Congruity Theory: Eagly and Karau 2002; Lack of Fit Theory: Heilman 2001). These classic theories are typically used to explain the underrepresentation or undervaluing of women in leadership roles (i.e., female leadership *disadvantage*).

The currently proposed personality-based explanatory model of the gender gap in transformational leadership supports the existence of two sets of countervailing theoretical mechanisms, articulating and supporting both a *female leadership advantage* (via emotional intelligence and communion) and a *female leadership disadvantage* (via agency). The current study also shows that these mechanisms not only help explain the gender gap in transformational leadership, but that the same mechanisms also explain the gender gap in leadership self-efficacy/self-rated transformational leadership.

### 4.2. Practical Implications

The double-bind (created by the role incongruity of gender roles and leader roles) for (potential) female leaders has been one mainstream concept used to describe the challenges women in leadership roles may face in contemporary organizations. In this female leadership narrative, women face the dilemma of choosing either to be agentic (congruent with their leader role, but incongruent with their stereotypical gender role), or choosing to be communal (incongruent with their leader role, but congruent with their stereotypical gender role). However, the results from the current study suggest that agency and communion are both beneficial to being perceived as a transformational leader. We echo that women and men need not choose between agency *or* communion (Bem 1974). Results from the current study suggest that agency and communion help people in general to be perceived as a leader—suggesting that one way to be perceived as a transformational leader is to be *both* assertive and compassionate.

The current study also found that emotional intelligence helps people to be perceived as a more transformational leader. Organizations hoping to strengthen their leadership could consider implementing emotional intelligence training (Cherniss and Adler 2000). Research using experimental designs has shown it is possible to increase emotional intelligence through training (Nelis et al. 2009), which could be one effective way to increase overall transformational leadership in organizations.

### 4.3. Limitations and Future Research Directions

First, the original meta-analyses in the current study focused exclusively on validated measurement instruments (e.g., EI was measured using performance-based ability EI measures only; leadership was measured using TFL measures only), in order to enhance construct validity. As a result of this attempt to strengthen construct validity, some of the original meta-analyses in the current study were based on a small number of primary studies/samples (k’s ranged from 4 to 17; N’s ranged from 420 to 1923). Future meta-analytic research could update the current results after more primary data accumulate, to increase the sample size.

Second, all of the primary studies included in the original meta-analyses in the current study were correlational studies (i.e., non-experimental designs). Accordingly, we could not draw causal inferences from the current study. Third, one limitation of all meta-analyses is that researchers can only study relationships that have been investigated by previous studies. In the current study, the agency-EI and communion-EI relationships were based on particularly small numbers of primary studies/samples, because little past research had examined the relationships between agency and EI, and between communion and EI.

With respect to future research, the current set of findings raises additional questions about role congruity theory (Eagly and Karau 2002). In particular, it is less clear that the leadership role is entirely masculine. Rather, the current set of results highlights that the transformational leadership role in particular may contain elements of communion/stereotypical femininity and emotional competence. The leadership role is multifaceted, in a manner that includes both stereotypically masculine and stereotypically feminine features simultaneously.

Additionally, the current design was unable to assess interaction effects. For instance, we could not assess whether agency predicted TFL better for men (see Kim et al. 2020), or whether communion and EI predicted TFL better for women (as implied by role congruity theory). Another limitation of the current study is that potential interaction effects between the mediators cannot be tested. For example, we could not test whether being both high in communion and EI at the same time would lead to an even greater leadership advantage. In other words, we could not test whether communion is more effective for leadership when people have high EI (i.e., a multiplicative effect of “will do” and “can do” communal attributes).

Methodologically, all meta-analytic SEM studies potentially have the issues of using a mixture of subpopulations (Newman et al. 2007). Therefore, it could be unclear whether results generalize to any particular subpopulation. Another common issue for such studies is construct validity. Almost always, multiple measurement instruments are used to measure one single construct. One effort we made to minimize construct validity concerns in the current study was to be selective with the measurement instruments by including primary studies with validated measures of emotional intelligence, communion, agency, and transformational leadership.

## 5. Conclusions

The current study showed that the gender gap in transformational leadership could be jointly explained by three personality-based mechanisms (i.e., emotional intelligence, communion, and agency). Specifically, emotional intelligence and communion could help explain the *female leadership advantage*, whereas agency could help explain a countervailing *female leadership disadvantage*. This set of findings expands upon Eagly’s (1987) social role theory and explanations (i.e., sex roles of agency/masculinity and communion/femininity) to also incorporate knowledge and skills-based tests of emotional intelligence (i.e., social and emotional skill) simultaneously in the exhibition of transformational leadership. Altogether, these three mechanisms, working in different directions, aggregate to a near-zero total gender gap in transformational leadership. The near-zero total effect hides a set of more substantial underlying gendered leadership phenomena (female leadership advantage based on emotional intelligence and communion, and male leadership advantage based on agency) that work in opposing directions to offset each other.

## Figures and Tables

**Table 1:** Meta-Analytic Correlation Matrix of Gender, Emotional Intelligence, Communion, Agency, and Transformational Leadership.

	1.	2.	3.	4.	5.
1. Gender	-				
2. Emotional Intelligence (performance-based ability EI measures only)	−.25 ^b^ (14/2216)	-			
3. Communion	−.30 ^c^ (937/254,465)	.07 ^a^ (5/1172)	-		
4. Agency	.23 ^c^ (928/254,731)	−.0005 ^a^ (5/1172)	.09 ^d^ (554/110,243)	-	
5. Transformational Leadership (non-self report)	−.09 ^e^ (10/2996)	.13 ^a^ (6/618)	.50 ^a^ (8/779)	.27 ^a^ (6/420)	-
6. Transformational Leadership (leader’s self-report)	−.15 ^e^ (10/836)	.29 ^a^ (17/1923)	.36 ^a^ (3/589)	.31 ^a^ (4/820)	.27 ^f^ (23/2784)

Note. Each cell contains *ρ* (the mean sample size-weighted meta-analytic correlation corrected for unreliability attenuation in both X and Y, but with no correction for the gender variable), followed by *k* (number of effect sizes) and *N* (total sample size), presented as (*k*/*N*). ^a^ Original meta-analysis. ^b^ Joseph and Newman (2010). ^c^ Hsu et al. (2021). ^d^ Badura et al. (2018). ^e^ Extracted from Eagly et al. (2003, pp. 576–77). ^f^ Lee and Carpenter (2018). Gender: F = 1, M = 2.

**Table 2:** Meta-analytic results for Emotional Intelligence and Transformational Leadership.

	*k*	*N*	*r*	*ρ*	*SD_ρ_*	95% CI	80% CV
Emotional Intelligence-Transformational leadership	28	2953	.20	.23	.16	[.14, .26]	[.03, .43]
**Rater**							
Self-Rated TFL	17	1923	.25	.29	.16	[.17, .33]	[.08, .50]
Other-Rated TFL	6	618	.12	.13	.02	[.04, .20]	[.11, .16]
Subordinate	5	472	.15	.17	.00	[.06, .23]	[.17, .17]
Mix of other raters (i.e., supervisor, peer, and subordinate)	1	146	.03	.03	.00	[.03, .03]	[.03, .03]
Mixed (e.g., self mixed with other)	5	412	.08	.09	.03	[−.02, .18]	[.05, .13]
**Sample type**							
Organizational sample	26	2419	.20	.24	.18	[.13, .27]	[.01, .46]
Other sample	2	534	.17	.21	.00	[.15, .20]	[.21, .21]
MBA working student sample	1	375	.16	.19	.00	[.16, .16]	[.19, .19]
Mixed sample (i.e., including both students and nonstudents)	1	159	.20	.23	.00	[.20, .20]	[.23, .23]
**Type of industry**							
Banking	1	138	.08	.09	.00	[.08, .08]	[.09, .09]
Education	7	521	.11	.13	.00	[.04, .18]	[.13, .13]
Welfare Compensation and Job Search Activities	1	102	.28	.32	.00	[.28, .28]	[.32, .32]
Manufacturing	1	133	.08	.09	.00	[.08, .08]	[.09, .09]
Hospitality	1	142	.22	.24	.00	[.22, .22]	[.24, .24]
Religious Organizations	1	27	.01	.01	.00	[.01, .01]	[.01, .01]
Mixed	8	703	.27	.32	.30	[.09, .46]	[−.06, .71]
**Sample country**							
US	10	937	.14	.16	.07	[.07, .21]	[.07, .25]
Other	12	1430	.22	.26	.21	[.11, .33]	[−.01, .53]
**Publication type**							
Published	7	824	.15	.18	.07	[.07, .23]	[.09, .27]
Unpublished	23	2333	.22	.26	.17	[.15, .29]	[.04, .47]

Note. *k* = number of effect sizes in the meta-analysis; *N* = total sample size in the meta-analysis; *r* = uncorrected correlation; *ρ* = correlation corrected for attenuation in the predictor and criterion; *SD_ρ_* = standard deviation of the corrected correlation; CI = confidence interval around *r*; CV = credibility interval around *ρ*.

**Table 3:** Meta-analytic results for Communion and Transformational Leadership.

	*k*	*N*	*R*	*ρ*	*SD_ρ_*	95% CI	80% CV
Communion-Transformational leadership	11	1368	.38	.44	.20	[.27, .50]	[.19, .69]
**Rater**							
Self-Rated TFL	3	589	.30	.36	.18	[.11, .50]	[.13, .59]
Other-Rated TFL	8	779	.44	.50	.19	[.32, .57]	[.26, .75]
Subordinate	8	779	.44	.50	.19	[.32, .57]	[.26, .75]
**Sample type**							
Organizational sample	10	1154	.37	.43	.21	[.24, .50]	[.15, .70]
Student sample	1	214	.45	.51	.00	[.45, .45]	[.51, .51]
**Type of industry**							
Banking	2	76	.62	.67	.00	[.59, .65]	[.67, .67]
Education	2	116	.31	.33	.30	[−.12, .75]	[−.06, .71]
Mixed	5	862	.35	.41	.21	[.18, .52]	[.14, .68]
**Sample country**							
US	4	548	.27	.31	.22	[.06, .49]	[.03, .59]
Other	5	628	.47	.53	.00	[.41, .52]	[.53, .53]
**Publication type**							
Published	7	820	.46	.53	.12	[.36, .55]	[.37, .68]
Unpublished	4	548	.27	.31	.22	[.06, .49]	[.03, .59]

Note. *k* = number of effect sizes in the meta-analysis; *N* = total sample size in the meta-analysis; *r* = uncorrected correlation; *ρ* = correlation corrected for attenuation in the predictor and criterion; *SD_ρ_* = standard deviation of the corrected correlation; CI = confidence interval around *r*; CV = credibility interval around *ρ*.

**Table 4:** Meta-analytic results for Agency and Transformational Leadership.

	*k*	*N*	*R*	*ρ*	*SD_ρ_*	95% CI	80% CV
Agency-Transformational leadership	10	1,240	.23	.30	.25	[.10, .37]	[−.02, .61]
**Rater**							
Self-Rated TFL	4	820	.25	.31	.15	[.11, .39]	[.12, .51]
Other-Rated TFL	6	420	.20	.27	.37	[.−06, .45]	[−.20, .74]
Subordinate	6	420	.20	.27	.37	[.−06, .45]	[−.20, .74]
**Sample type**							
Organizational sample	9	1026	.21	.27	.27	[.06, .36]	[−.07, .61]
Student sample	1	214	.36	.42	.00	[.36, .36]	[.42, .42]
**Type of industry**							
Banking	2	76	.43	.49	.00	[.42, .43]	[.49, .49]
Education	1	52	.42	.46	.00	[.42, .42]	[.46, .46]
Mixed	4	567	.32	.42	.16	[.19, .45]	[.21, .63]
**Sample country**							
US	3	484	.36	.43	.00	[.33, .43]	[.43, .43]
Other	5	564	.10	.13	.25	[−.10, .31]	[−.19, .45]
**Publication type**							
Published	7	756	.15	.21	.29	[−.03, .34]	[−.17, .58]
Unpublished	3	484	.36	.43	.00	[.33, .38]	[.43, .43]

Note. *k* = number of effect sizes in the meta-analysis; *N* = total sample size in the meta-analysis; *r* = uncorrected correlation; *ρ* = correlation corrected for attenuation in the predictor and criterion; *SD_ρ_* = standard deviation of the corrected correlation; CI = confidence interval around *r*; CV = credibility interval around *ρ*.

**Table 5:** Meta-analytic results for Emotional Intelligence with Agency and Communion.

	*k*	*N*	*R*	*ρ*	*SD_ρ_*	95% CI	80% CV
**Emotional intelligence**							
Communion	5	1172	.05	.07	.15	[−.06, .16]	[−.12, .25]
Agency	5	1172	−.0009	−.0005	.00	[−.05, .05]	[−.0005, −.0005]

Note. *k* = number of effect sizes in the meta-analysis; *N* = total sample size in the meta-analysis; *r* = uncorrected correlation; *ρ* = correlation corrected for attenuation in the predictor and criterion; SD*_ρ_* = standard deviation of the corrected correlation; CI = confidence interval around *r*; CV = credibility interval around *ρ*.

**Table 6:** Fit statistics for alternative theoretical models.

Models	χ^2^	*df*	Δχ^2^	RMSEA	CFI	ΔCFI	NNFI	SRMR
**Model 1a**^a,c^: Gender-EI-Communion-Agency-TFL (Full Mediation)	.434	1	--	.0000	1.000	--	1.023	.0061
Model 1b ^a,d^: Gender-EI-Communion-Agency-TFL (Partial Mediation)	0 (saturated model)	0	.43	.0000	1.000	.000	1.000	.0061
								
**Model 2a**^b,c^: 2 DVs (Self and Other TFL): Gender-EI-Communion-Agency (Full Mediation)	2.513	2	--	.0247	.999	--	.990	.0127
Model 2b ^b,d^: 2 DVs (Self and Other TFL): (add direct path from Gender to Other-rated TFL)	2.073	1	.44	.0506	.997	.002	.958	.0116
Model 2c ^b,e^: 2 DVs (Self and Other TFL): (add direct path from Gender to Self-rated TFL)	.434	1	2.08	.0000	1.000	.001	1.022	.0052
Model 2d ^b,f^: 2 DVs (Self and Other TFL): (add 2 direct paths from Gender to Self- and Other-rated TFL)	0 (saturated model)	0	2.51	.0000	1.000	.001	1.000	.0000

Note. Models judged to fit best are in boldface. ΔCFI > .01 (Cheung and Rensvold 2002). Analyses based on minimum *N* = 420. ^a^ Includes only non-self report transformational leadership as the DV. ^b^ Includes both non-self and self-report transformational leadership as two DVs. ^c^ Does not include any direct paths from gender to any transformational leadership outcome variables. ^d^ Includes a direct path from gender to non-self-report transformational leadership. ^e^ Includes a direct path from gender to self-report transformational leadership. ^f^ Includes direct paths from gender to both non-self and self-report transformational leadership.

**Table 7:** Test of indirect effects for gender and transformational leadership (non-self-reported TFL and self-reported TFL).

Path	Product of Coefficients	Indirect Effect	95% Monte Carlo CI	Statistically Significant
Gender→Emotional Intelligence→TFL (non-self) ^a^	(−.250)*(.097)	−.0243	[−.0483, −.0041]	Yes
Gender→Communion→TFL (non-self) ^a^	(−.300)*(.473)	−.1419	[−.1945, −.0948]	Yes
Gender→Agency→TFL (non-self) ^a^	(.230)*(.228)	.0524	[.0268, .0837]	Yes
Gender→Emotional Intelligence→TFL (self-report) ^b^	(−.250)*(.268)	−.0670	[−.1012, −.0376]	Yes
Gender→Communion→TFL (self-report) ^b^	(−.300)*(.316)	−.0948	[−.1361, −.0597]	Yes
Gender→Agency→TFL (self-report) ^b^	(.230)*(.282)	.0649	[.0348, .0998]	Yes

Note. ^a^ Indirect effects tests are based on path coefficients from Model 1a (non-self-reported TFL), and are also identical to the path coefficients from Model 2a for non-self-reported TFL. ^b^ Indirect effects tests are based on path coefficients from Model 2a (self-reported TFL). TFL = transformational leadership.

## Data Availability

The data presented in this study are available from the first author.
